# Real-world effectiveness and persistence of secukinumab in the treatment of patients with psoriatic arthritis

**DOI:** 10.3389/fmed.2023.1294247

**Published:** 2023-11-20

**Authors:** Juan José Alegre-Sancho, Victoria Núñez-Monje, Cristina Campos-Fernández, Isabel Balaguer-Trull, Montserrat Robustillo-Villarino, Marta Aguilar-Zamora, Marta Garijo-Bufort, Teresa Pedraz-Penalva, Carolina Peña-González, Isabel de la Morena, Diego Bedoya-Sanchís, Liliya Yankova-Komsalova, Arantxa Conesa-Mateos, Anna Martinez-Cristóbal, Francisco Javier Navarro-Blasco, Jose Miguel Senabre-Gallego, Francisca Sivera

**Affiliations:** ^1^Rheumatology Department, Hospital Universitario Doctor Peset, Valencia, Spain; ^2^Rheumatology Department, Hospital General Universitario, Valencia, Spain; ^3^Rheumatology Department, Hospital Universitario de la Plana, Villareal, Spain; ^4^Rheumatology Department, Hospital de Sagunto, Valencia, Spain; ^5^Rheumatology Department, Hospital General Universitario de Elda, Elda, Spain; ^6^Rheumatology Department, Hospital Francesc De Borja, Gandía, Spain; ^7^Rheumatology Department, Hospital Clínico Universitario De Valencia, Valencia, Spain; ^8^Rheumatology Department, Hospital de Dénia-Marina Salud, Dénia, Spain; ^9^Rheumatology Department, Hospital General Universitario de Castellón, Castellón de la Plana, Spain; ^10^Rheumatology Department, Hospital Universitario La Ribera, Alzira, Spain; ^11^Rheumatology Department, Hospital General Universitario de Elche, Elche, Spain; ^12^Rheumatology Department, Hospital Marina Baixa, Villajoyosa, Spain

**Keywords:** secukinumab, psoriatic arthritis, real-world evidence, effectiveness, persistence

## Abstract

**Introduction:**

Psoriatic arthritis (PsA) is a complex and heterogeneous inflammatory disease. Secukinumab, a biologic disease-modifying antirheumatic drug (bDMARD), has extensive clinical evidence of efficacy and safety in the treatment of PsA but data in clinical practice are still limited. This study aims to provide real-world evidence on secukinumab use, effectiveness, and persistence in PsA.

**Methods:**

A retrospective, multicenter study was conducted on patients diagnosed with PsA and treated with secukinumab up to June 2021 at 12 centers in the Valencian Community (Spain). Data on DAS28-CRP, DAPSA, Tender and Swollen Joint Counts (TJC, SJC), enthesitis, dactylitis, skin and nail involvement, pain, patient and physician global assessment (ptGA, phGA) using 100-mm visual analog scale (VAS), and persistence for up to 24 months were collected.

**Results:**

A total of 178 patients were included (49% men; mean [standard deviation, SD] age: 51.4 [10.5] years; 39% obese). Secukinumab was used as a first-, second-, or ≥ third-line bDMARD in 37, 21, and 42% of patients, respectively. The percentage of patients achieving at least low disease activity (DAS28-CRP ≤ 3.2) increased from 25% at baseline to 66% at month 6 (M6) and was maintained (75%) up to M24. Mean (SD) DAS28-CRP baseline values (3.9 [1.2]) decreased to 2.9 (1.1) (*p* < 0.001) at M6 and remained low through M24 (2.6 [1.1]) (*p* < 0.001). Secukinumab also improved peripheral arthritis increasing the percentage of patients with TJC = 0 (20% baseline; 57% M24) and SJC = 0 (37% baseline; 80% M24). Treatment reduced the percentage of patients with enthesitis (25% baseline; 6% M24), dactylitis (20% baseline; 4% M24), and skin (70% baseline; 17% M24), and nail (32% baseline; 2% M24) involvement. Additionally, we observed improvements in the mean pain VAS (−26.4 mm M24), ptGA (−26.2 mm M24), and phGA (−24.8 mm M24). Secukinumab showed an overall 24-month persistence rate of 67% (95% confidence interval [CI]: 60–74%). Patients receiving first-line secukinumab showed the highest 24-month persistence rate (83, 95% CI: 73–92; *p* = 0.024).

**Conclusion:**

Secukinumab showed long-term effectiveness across the six key PsA domains thus reducing disease activity and pain, which are major treatment goals. This was accompanied by high persistence rates, especially in bDMARD naive patients.

## 1 Introduction

Psoriatic arthritis (PsA) is a chronic inflammatory disease of the peripheral and axial skeleton affecting 0.1–0.25% of the world population (1, 2) and up to 30% of patients with psoriasis (3, 4). It is a highly heterogeneous disorder that may result from distinct immune mechanisms, and can present as peripheral arthritis, axial disease, enthesitis, dactylitis, nail dystrophy, and skin psoriasis, making it difficult to diagnose (5). In addition, PsA is frequently associated with conditions and comorbidities such as uveitis, inflammatory bowel disease (IBD), obesity, metabolic syndrome, and cardiovascular disease (6–8). PsA can cause irreversible joint destruction and deformity, which can profoundly impair the patient’s quality of life (QoL) (9, 10). Therefore, prompt diagnosis and treatment are of the utmost importance in PsA patients.

Assessing patients with PsA requires a multidisciplinary approach (11, 12). International treatment guidelines recommend evaluating disease activity in each of the PsA domains as well as considering comorbidities, previous therapies, and patient preferences. When possible, treatment should be selected to address all active domains and any related conditions (8). Since most patients have multiple affected domains at presentation (13), it is essential to identify the most severely affected domains, personalize treatment choices, and periodically re-evaluate patients (8).

According to the updated GRAPPA recommendations for the treatment of PsA, topical agents are strongly recommended as the first-line treatment for psoriasis, and conventional synthetic disease-modifying anti-rheumatic drugs (csDMARDs)—such as methotrexate (MTX), sulfasalazine, cyclosporine, and leflunomide—are recommended for arthritis (14). While these drugs are effective in relieving symptoms and in treating peripheral arthritis, they exhibit limited efficacy in slowing radiographic progression, reducing axial symptoms, or improving uveitis, enthesitis, and dactylitis (15). Therefore, biologic DMARDs (bDMARDs) and targeted synthetic DMARDs (tsDMARDs) can be considered as first-line therapies in many instances (8).

Within bDMARDs, tumor necrosis factor inhibitors (TNFi) and IL-17 inhibitors (IL-17i) have demonstrated efficacy in treating multiple domains of PsA, including peripheral and axial arthritis, enthesitis, dactylitis, psoriasis, and nail disease, and in reducing radiographic progression (16–18). TNFi and IL-17i have shown comparable efficacies in peripheral arthritis, with both recommended as alternatives in patients with inadequate response to csDMARDs (19). Similarly, both bDMARD types are strongly recommended in patients presenting with enthesitis, dactylitis, nail psoriasis, and axial disease (8). In fact, IL-17i and TNFi constitute the only strongly recommended bDMARDs for all key domains of PsA, therefore being especially relevant when multiple domains are affected. Moreover, IL-17i have shown to be more effective than TNFi in treating skin involvement in psoriasis and/or PsA patients (8, 19, 20).

Secukinumab (Cosentyx®) is a recombinant fully human monoclonal IgG1/k antibody that targets the pro-inflammatory cytokine IL-17A, inhibiting its interaction with the receptor, which reduces the inflammatory effects mediated by IL-17A (21, 22). Secukinumab was approved by the European Medicines Agency in January 2015 for the treatment of PsA in adult patients when the response to previous DMARD therapy has been inadequate (23). In two-phase III clinical trials, FUTURE-1 and FUTURE-2, secukinumab was demonstrated to be safe and better than placebo in improving disease activity at 24 weeks (24, 25). These studies also showed that secukinumab is effective in both TNFi-naive and TNFi-experienced patients, with the 300 mg dose being especially effective in patients previously exposed to TNFi (26). The 5-year results of FUTURE-1 and FUTURE-2 clinical trials showed sustained improvement of signs and symptoms of PsA, with consistent safety (27, 28). Notably, in the phase III clinical trial MAXIMISE, secukinumab demonstrated significant improvements in axial disease signs and symptoms (29); so far, MAXIMISE is the only study providing data on axial PsA. Finally, the FUTURE-5 study showed that secukinumab was able to inhibit radiographic structural progression through 2 years in patients with PsA (30, 31).

Despite the extensive variety of randomized clinical trials (RCTs) that demonstrate the efficacy and safety of secukinumab, these studies include highly selected patient populations and may not be applicable to the general PsA population. Real-world data (RWD) complement clinical trial data by gathering routine clinical practice, which encompasses a broader spectrum of patients (32). Even with international efforts such as the SERENA and EuroSpA studies (33, 34), real-world evidence on secukinumab use in patients with PsA is currently limited, highlighting the need to conduct RWD studies. This study aims to analyze the effectiveness and persistence of secukinumab in patients with PsA in a real clinical setting.

## 2 Methods

### 2.1 Study design and patient characteristics

This was an observational, retrospective, and multicenter study in patients with PsA. Adult patients (≥18 years old) with a diagnosis of PsA, as per their treating physician, and who were receiving or had received treatment with secukinumab up to June 2021, were included in the study. Patients were excluded if they had received secukinumab in the context of a clinical trial or as off-label treatment. Data from the Rheumatology departments of 12 hospitals in the Valencian Community (Spain) were retrospectively collected from secukinumab initiation and every 6 months.

The study was approved by the Ethics Committee of the General University Hospital in Elda (Alicante, Spain). The study was conducted in accordance with the ethical principles of the Declaration of Helsinki, Good Clinical Practice (GCP), and in compliance with European and local requirements. Written informed consent was not required in accordance with the national legislation (Real Decreto 957/2020).

### 2.2 Study endpoints

The demographic, anthropometric, and clinical profiles of patients diagnosed with PsA were recorded. The number of patients who started secukinumab as first bDMARD (naive) or who received prior bDMARD treatment (second or ≥ third line) as well as secukinumab starting dose (150 or 300 mg) and uptitration data were collected.

Effectiveness was assessed, from baseline to 24 months after secukinumab initiation in 6-monthly intervals, by DAS28-CRP [C-reactive protein-based DAS28 (count of 28 swollen and tender joints)], Disease Activity in PSoriatic Arthritis (DAPSA), Tender Joint Count (TJC), and Swollen Joint Count (SJC; using the 28-joint count), presence of skin and nail involvement, presence of enthesitis and dactylitis, and CRP (mg/L). In addition, perception of health was evaluated by patient’s pain, patient’s global assessment (ptGA), and physician’s global assessment (phGA) of the disease, using visual analog scales (VAS; from 0 to 100, with higher scores indicating worse pain/disease activity). Secukinumab persistence at 24 months and reasons for discontinuation were also collected.

### 2.3 Data analysis

Measures of central tendency and dispersion are presented [mean and standard deviation (SD) or median and interquartile range (IQR)] for continuous variables, and frequencies and percentages for categorical variables to describe the use of secukinumab in patients with PsA, the profile of these patients and secukinumab efficacy and persistence; 95% confidence intervals (CI) were calculated.

Secukinumab persistence was analyzed by using the Kaplan–Meier method. The persistence of secukinumab was quantified as the time from the start of secukinumab until the end of treatment (definitive discontinuation) or until the end of data collection in those patients who continued on treatment. Differences between groups (naive, second line, third or posterior lines) in secukinumab persistence were analyzed using the log-rank test.

## 3 Results

### 3.1 Patient characteristics

A total of 178 patients diagnosed with PsA met the selection criteria and were enrolled in the study. As shown in Table 1, the mean (SD) age was 51.4 (10.5) years old (range: 23.5–75.2) and 49% were men. The median (IQR) time to PsA diagnosis was 0.7 (0.3–2.1) years. Also, 57% of patients presented at least one comorbidity, mainly dyslipidemia (57%) and hypertension (56%). At secukinumab initiation, 70 and 32% of patients showed skin and nail lesions, respectively. Peripheral arthritis was present in 90% of patients; TJC (1–28) and SJC (1–28) mean (SD) were 6.3 (5.5) and 3.4 (2.3), respectively. Thirty-nine percent of patients presented with axial disease. A smaller number of patients presented with enthesitis (25%) or dactylitis (20%). Mean (SD) scores at baseline (before starting secukinumab treatment) were: DAS28-CRP 3.9 (1.1), DAPSA 20.3 (9.4), CRP 4.6 (10) mg/dL, pain VAS 58.4 (25.1), ptGA 56.5 (26.1), and phGA 46.4 (24).

**Table 1:** Baseline characteristics of the population.

Parameter of study	
Sex (male), *n* (%) [*N*]	87 (49) [178]
Ethnicity (Caucasian), *n* (%) [*N*]	170 (96) [178]
Age, mean (SD) [*N*]	51.4 (10.5) [178]
BMI (kg/m^2^), mean (SD) [*N*]	28.8 (4.7) [108]
Obese (BMI ≥ 30), *n* (%) [*N*]	42 (39) [108]
Current smokers, *n* (%) [*N*]	54 (35) [154]
Age at symptoms initiation, mean (SD) [*N*]	41.7 (11.2) [137]
Years from first symptoms to diagnosis, median (IQR) [*N*]	0.7 (0.3–2.1) [137]
Years from diagnosis to SEC treatment, median (IQR) [*N*]	6.2 (2.4–10) [170]
Axial disease, *n* (%) [*N*]	70 (39) [178]
Peripheral arthritis, *n* (%) [*N*]	160 (90) [178]
Polyarticular, *n* (%) [*N*]	70 (44) [160]
Oligoarticular, *n* (%) [*N*]	76 (48) [160]
Enthesitic, *n* (%) [*N*]	14 (9) [160]
TJC (1–28), mean (SD) [*N*]	6.3 (5.5) [104]
TJC ≥ 1, *n* (%) [*N*]	104 (80) [130]
SJC (1–28), mean (SD) [*N*]	3.4 (2.3) [82]
SJC ≥ 1, *n* (%) [*N*]	82 (63) [130]
Enthesitis (yes), *n* (%) [*N*]	36 (25) [146]
Number, mean (SD) [*N*]	2.1 (1.3) [31]
Dactylitis (yes), *n* (%) [*N*]	30 (20) [148]
Number, mean (SD) [*N*]	3.1 (2.3) [24]
Skin involvement (yes), *n* (%) [*N*]	114 (70) [162]
Nail involvement (yes), *n* (%) [*N*]	50 (32) [157]
Uveitis (yes), *n* (%) [*N*]	3 (2) [169]
DAS28-CRP, mean (SD) [*N*]	3.9 (1.1) [100]
DAPSA, mean (SD) [*N*]	20.3 (9.4) [33]
Pain VAS, mean (SD) [*N*]	58.4 (25.1) [111]
ptGA, mean (SD) [*N*]	56.5 (26.1) [82]
phGA, mean (SD) [*N*]	46.4 (24) [66]
CRP (mg/dl), mean (SD) [*N*]	4.6 (10) [162]
HLA-B27 (positive), *n* (%) [*N*]	24 (20) [121]
Prior anti-bDMARDs (%) [*N*]	113 (68) [166]
Comorbidities (any), *n* (%) [*N*]	101 (57) [178]
Dyslipidemia, *n* (%) [*N*]	58 (57) [101]
Hypertension, *n* (%) [*N*]	57 (56) [101]
Depression, *n* (%) [*N*]	37 (37) [101]
Diabetes, *n* (%) [*N*]	27 (27) [101]
Hepatic steatosis, *n* (%) [*N*]	22 (22) [101]
Tuberculosis/latent tuberculosis infection, *n* (%) [*N*]	19 (19) [101]
Cardiovascular events, *n* (%) [*N*]	13 (13) [101]
Neoplasms, *n* (%) [*N*]	4 (4) [101]

bDMARD, Biologic disease-modifying antirheumatic drug; BMI, Body mass index; CRP, C reactive protein; IQR, Interquartile range; psGA, Physician global assessment; ptGA, Patient global assessment; SD, Standard deviation; SEC, Secukinumab; SJC, Swollen joint count; TJC, Tender joint count; VAS, Visual analog scale.

### 3.2 Treatment with secukinumab

**Figure 1:**
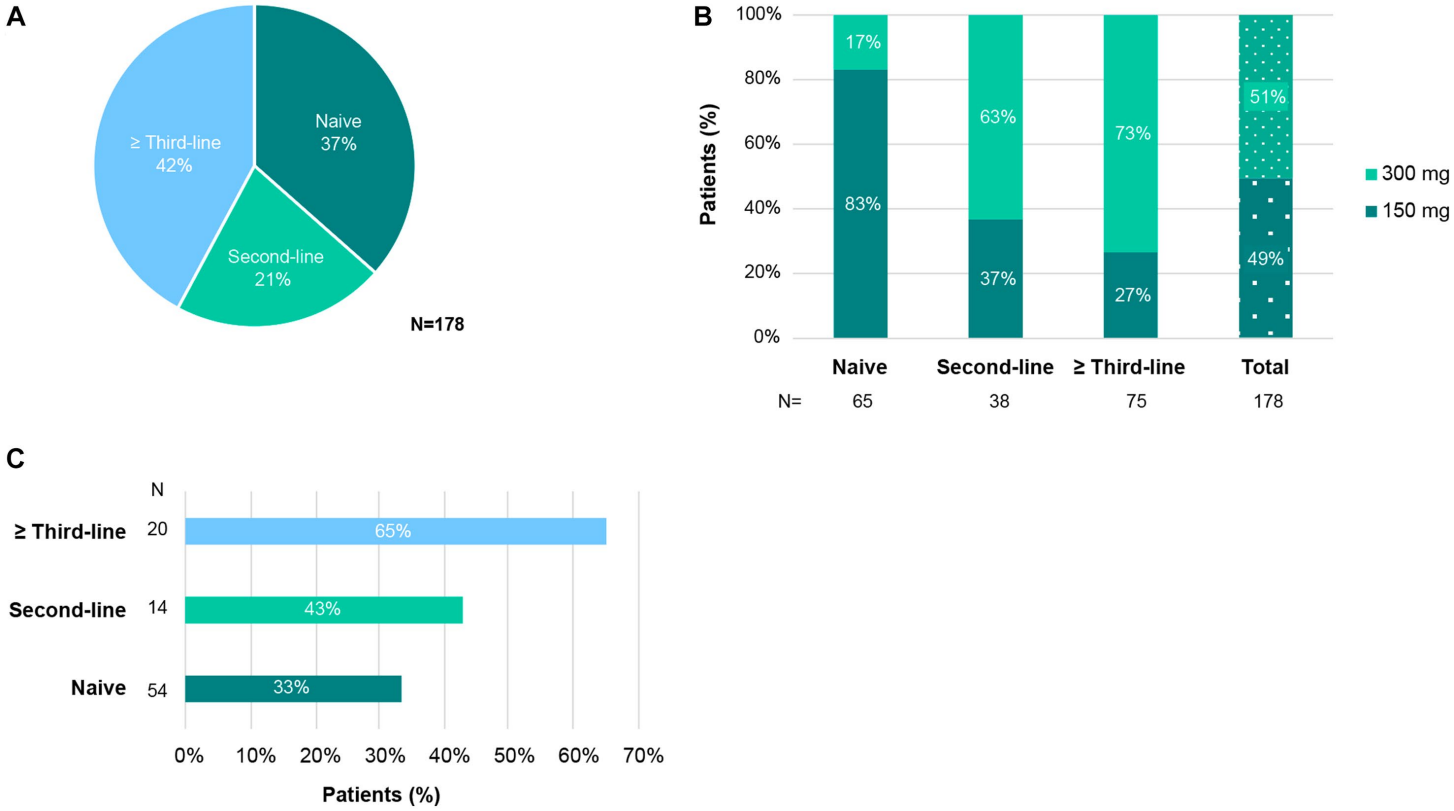
Use of secukinumab in patients with PsA. **(A)** Percentage of patients that started secukinumab as first-line (naive), second-line, or ≥ third-line bDMARD. **(B)** Percentage of patients who received secukinumab 150 or 300 mg initiation dose, by previous exposure to bDMARDs. **(C)** Percentage of patients treated with 150 mg secukinumab that uptitrated to 300 mg, per bDMARD treatment line.

Patients initiated treatment with secukinumab on a median (IQR) of 6.2 (2.4–10) years after PsA diagnosis. Prior to secukinumab, 84% had been treated with a csDMARD, 64% with a bDMARD and 6% with a tsDMARD (Supplementary Table 1). As shown in Figure 1A, 37% of patients initiating secukinumab treatment were naive while 21 and 42% of patients received secukinumab as the second or third and posterior-lines of treatment, respectively. Forty-seven percent of patients initiated secukinumab treatment as monotherapy and 53% in combination with csDMARDs. Regarding secukinumab initial dose, 83% of naive patients started treatment with the 150 mg dose while 63% of second-line and 73% of third or posterior-line patients started with the 300 mg dose (Figure 1B). Nevertheless, as shown in Figure 1C, 33% of naive, 43% of second-line, and 65% of third or posterior-line patients who started with the 150 mg dose up-titrated to 300 mg. Mean (SD) duration of secukinumab treatment was 23.7 (17.1) months.

### 3.3 Effectiveness of secukinumab treatment

**Figure 2:**
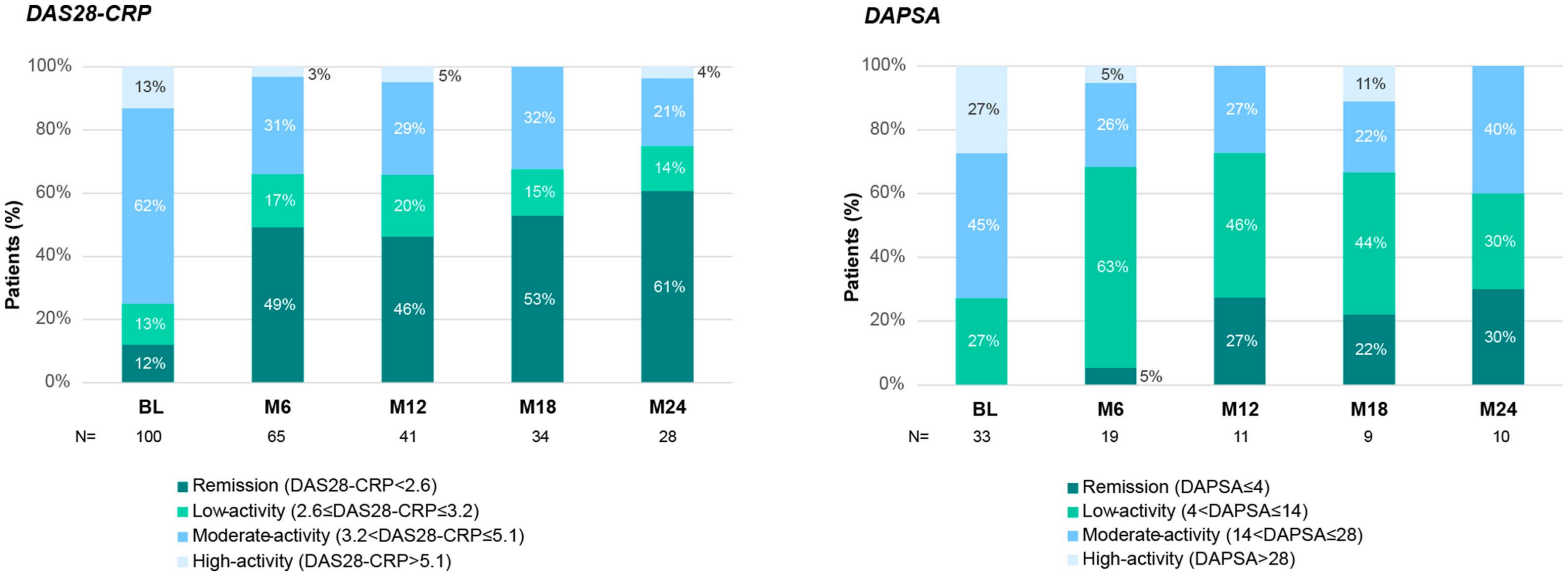
Disease activity during secukinumab treatment. Disease activity measured by DAS28-CRP (left panel) and DAPSA (right panel). Stacked columns show the percentage of patients in remission or with low-, moderate-, or high-disease activity at each time point, throughout the 24-month follow-up with secukinumab treatment. BL, Baseline; M6–M24, Month 6–24.

As observed in Figure 2, secukinumab was able to increase the percentage of patients in remission or low disease activity in the different indices measured. According to DAS28-CRP, after 6 months of treatment, the proportion of patients with at least low disease activity increased from 25 to 66% and the effect was maintained up to 24 months. In fact, 49% of patients achieved the stringent criteria of remission at 6 months (baseline: 12%). Regarding DAPSA, the results were similar, with 68% of patients achieving at least low activity of the disease by month 6 (baseline: 27%) and maintaining the effect it up to 24 months (60%). In addition, DAS28-CRP mean (SD) baseline values (3.9 [1.2]) decreased to 2.9 (1.1) at month 6 and remained low through month 12 (2.9 [1.2]) and month 24 (2.6 [1.1]) (*p* < 0.001 for all values in comparison to baseline).

**Figure 3:**
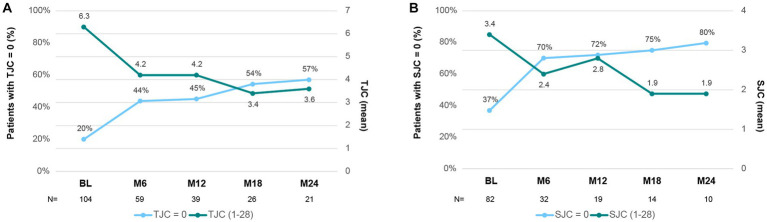
Tender (TJC) and Swollen (SJC) joint count during secukinumab treatment. **(A)** Mean TJC (1–28) at each time point (dark green). The percentage of patients with a TJC = 0 at each time point is represented in blue. **(B)** Mean SJC (1–28) at each time point (dark green). The percentage of patients with a SJC = 0 at each time point is represented in blue. BL, Baseline; M6–M24, Month 6–24.

Secukinumab also improved other items assessing peripheral arthritis, as shown in Figure 3. The percentage of patients with TJC = 0 improved from baseline (20%) up to 44% at month 6 and 57% at month 24. Of note, among patients not achieving TJC = 0, the mean (SD) TJC decreased from 6.3 (5.5) at baseline to 3.6 (3.0) at the end of follow-up (Figure 3A). Similarly, the percentage of patients with SJC = 0 increased from 37% at baseline, to 70% during the first 6 months and up to 80% at month 24. Mean (SD) SJC of patients remaining with at least SJC ≥ 1 also showed a marked decrease from baseline (3.4 [2.3]) to month 6 (2.4 [2.3]) and month 24 (1.9 [1.1]) (Figure 3B).

**Figure 4:**
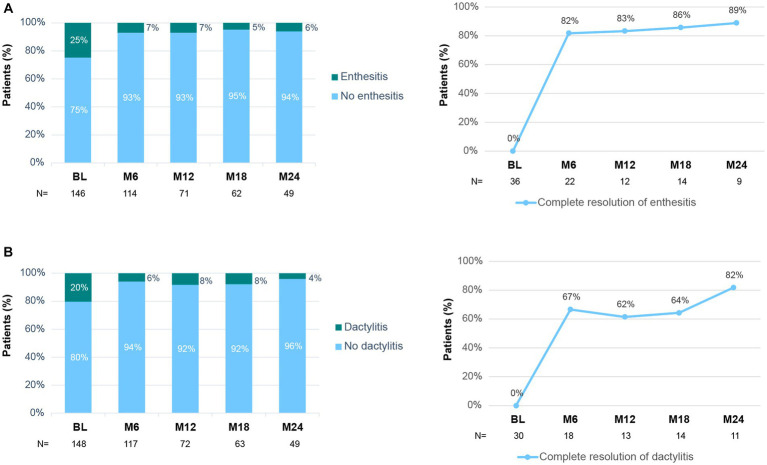
Enthesitis and dactylitis during secukinumab treatment. **(A)** Percentage of patients with/without enthesitis at each time point (left panel). Percentage of patients with baseline enthesitis that achieved complete resolution (right panel). **(B)** Percentage of patients with/without dactylitis at each time point (left panel). Percentage of patients with baseline dactylitis that achieved complete resolution (right panel). BL, Baseline; M6–M24, Month 6–24.

As shown in Figure 4A, the percentage of patients with enthesitis declined during secukinumab treatment, from 25% at baseline to 6% at month 24. Indeed, 82% of patients with baseline enthesitis achieved complete resolution after 6 months of treatment. The percentage of patients with dactylitis rapidly decreased from baseline to month 6 and remained stable up to 24 months of treatment (20% at baseline, 6% at month, and 4% at month 24; Figure 4B). A complete resolution of dactylitis was observed at month 6 in 67% of patients with reported baseline dactylitis.

**Figure 5:**
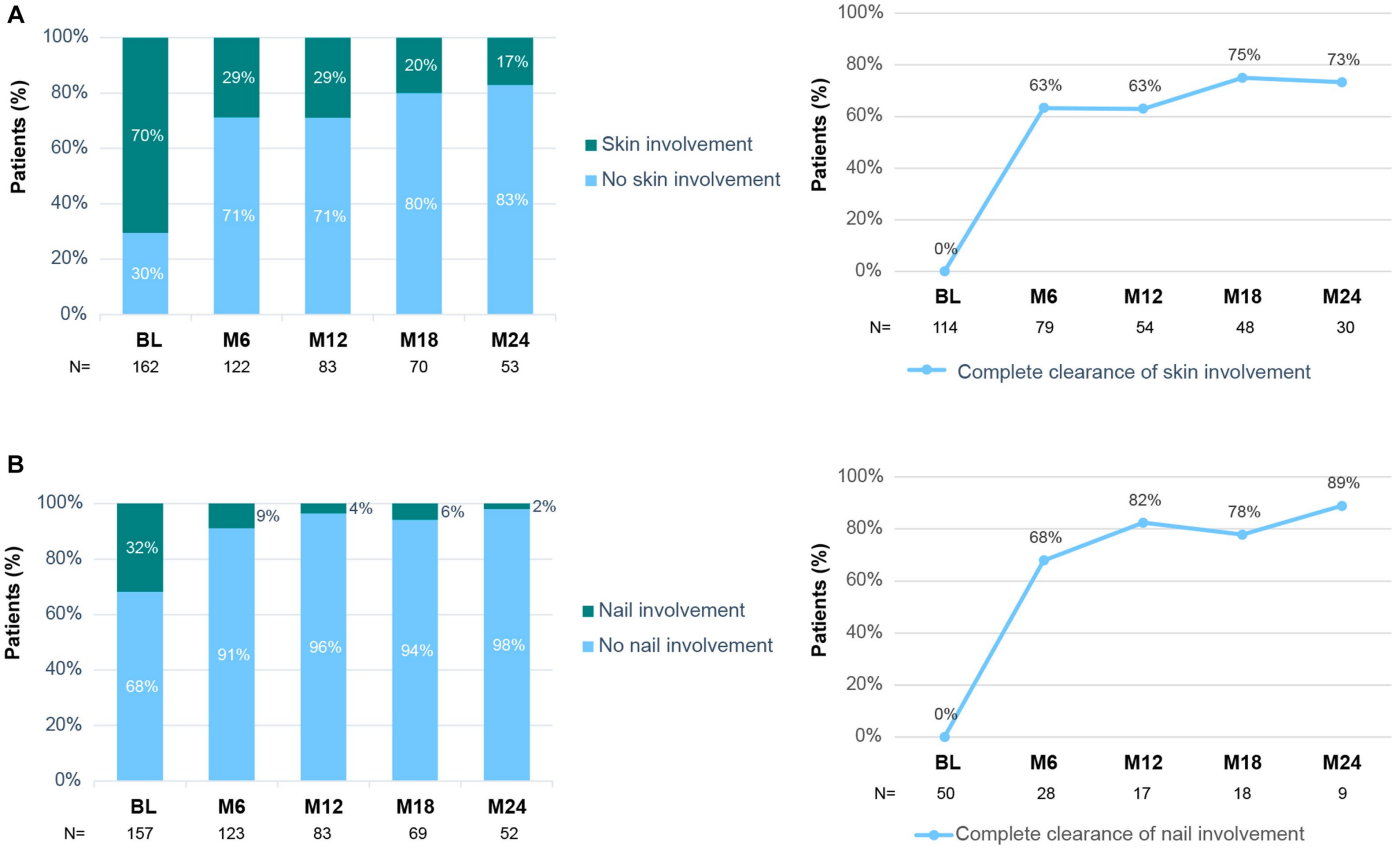
Skin and nail involvement during secukinumab treatment. **(A)** Percentage of patients with/without skin involvement at each time point (left panel). Percentage of patients with baseline skin involvement that achieved complete clearance (right panel). **(B)** Percentage of patients with/without nail involvement at each time point (left panel). Percentage of patients with baseline nail involvement that achieved complete clearance (right panel). BL, Baseline; M6–M24, Month 6–24.

Treatment with secukinumab also led to improvement in skin and nail lesions of the disease (Figure 5). The percentage of patients with skin involvement decreased from 70% at baseline to 29% at month 6 and 17% at month 24 (Figure 5A). In fact, 63% of patients with baseline skin lesions achieved a complete clearance after 6 months of secukinumab treatment (Figure 5A). Similarly, the proportion of patients with nail involvement decreased from 32% at baseline to 9 and 2% at 6 and 24 months, respectively. In accordance, 68% of patients achieved complete clearance of nail involvement at month 6 (Figure 5B). The levels of CRP were within the normal range at baseline and remained within that range throughout the study (data not shown).

**Figure 6:**
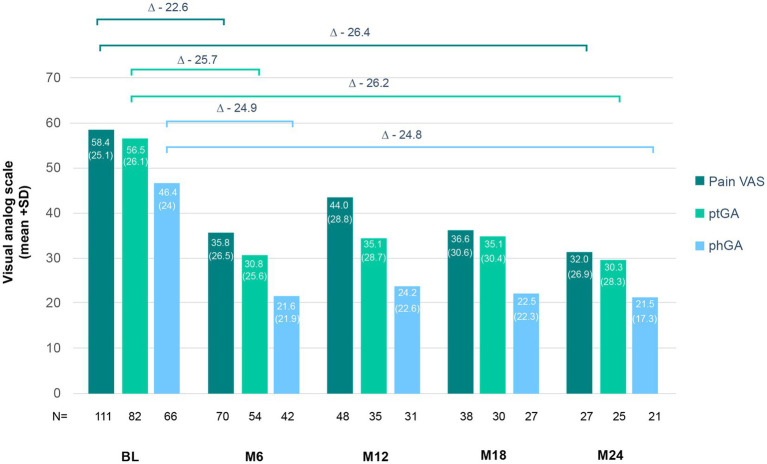
Changes in pain, ptGA, and phGA scores under secukinumab treatment. The mean + standard deviation (in brackets) of the patient’s pain, ptGA and phGA are represented for each time point. Delta represent mean changes from baseline (BL) to month 6 (M6) and month 24 (M24).

Furthermore, the impact of secukinumab on pain, ptGA, and phGA was also analyzed. Mean (SD) baseline pain VAS improved from 58.4 (25.1) to 35.8 (26.5) after 6 months of treatment and remained stable (32 [26.9]) up to month 24 (Figure 6). Mean (SD) ptGA also decreased from 56.5 (26.1) at baseline to 30.8 (25.6) at month 6 and 30.3 (28.3) at month 24. Similarly, phGA improved at month 6 and was maintained thereafter (46.4 [24] at baseline, 21.5 [17.3] at month 24; Figure 6).

### 3.4 Persistence

Secukinumab had an overall 12-month persistence rate of 78% (95% CI: 71–84) and a 24-month persistence rate of 67% (95% CI: 60–74; Figure 7). The highest 24-month persistence rate was observed in naive patients (83%; 95% CI: 73–92), followed by patients receiving secukinumab treatment as second (62%; 95% CI: 44–80), and third or posterior line (58%; 95% CI: 46–69; Figure 7).

**Figure 7:**
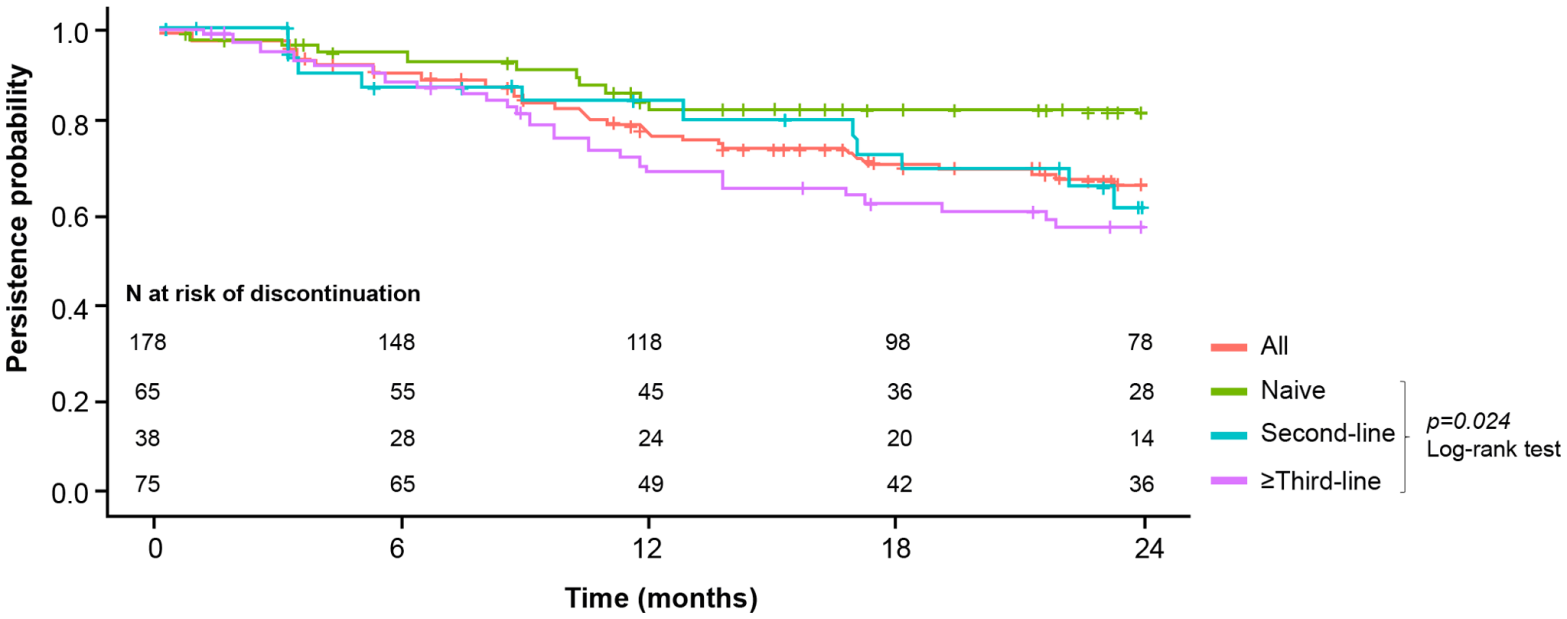
Secukinumab persistence per bDMARD treatment line. Accumulated persistence of secukinumab was calculated by the Kaplan–Meier method, considering the starting time of secukinumab and its discontinuation during 24 months of follow-up.

As shown in Table 2, 51 patients (29%) discontinued secukinumab treatment. Among them, reasons for discontinuation were mainly primary failure (45%), secondary failure (35%), adverse events (10%), other medical reasons (4%), intolerance (2%), loss of follow-up (2%), and patient decision (2%).

**Table 2:** Reasons for discontinuation of secukinumab treatment in PsA patients.

Causes for SEC discontinuation	
SEC discontinuation (any), *n* (%) [*N*]	51 (29) [178]
Primary failure, *n* (%) [*N*]	23 (45) [51]
Secondary failure, *n* (%) [*N*]	18 (35) [51]
Intolerance, *n* (%) [*N*]	1 (2) [51]
Adverse events, *n* (%) [*N*]	5 (10) [51]
Loss of follow-up, *n* (%) [*N*]	1 (2) [51]
Patient decision, *n* (%) [*N*]	1 (2) [51]
Other medical reasons, *n* (%) [*N*]	2 (4) [51]

SEC, Secukinumab.

## 4 Discussion

The main goal in the treatment of PsA is to achieve the lowest disease activity possible by controlling symptoms, preventing structural damage, optimizing physical function, and improving the patients’ QoL (35, 36). The present study provides real-world evidence of secukinumab in 178 patients with PsA treated at 12 rheumatology departments (Valencian Community, Spain). This study shows that secukinumab is able to rapidly reduce disease activity measured by both DAS28-CRP and DAPSA. Treatment with secukinumab demonstrated substantial improvements in key PsA domains, including peripheral arthritis, enthesitis, dactylitis, and skin and nail involvement. In accordance, pain VAS, ptGA and phGA scores also improved and, persistence of secukinumab was high for up to 24 months.

In the FUTURE trials, both 150 and 300 mg secukinumab doses showed a significantly higher proportion of patients achieving ≥20% improvement in the American College of Rheumatology response criteria (ACR20) in comparison to placebo, at week 16 (30, 37) and week 24 (24, 25, 38). Likewise, changes from baseline in DAS28-CRP were significantly greater with secukinumab compared to placebo (24, 25, 30, 37, 38). In our study, most (75%) patients presented at least moderate activity of the disease at baseline. Although 12% of patients were in DAS28-CRP remission at baseline, the indication for starting secukinumab in these patients was due to disease activity in other PsA domains and or safety/intolerance issues with the previous treatment. We observed that after 6 months of treatment the percentage of patients achieving at least low disease activity (DAS28-CRP ≤ 3.2) increased to 66%, which was maintained, or even slightly increased at 24 months (75%). In fact, 49% of patients were in remission (DAS28-CRP ≤ 2.6) at month 6 and this proportion increased up to month 24 (61%); this proportion is slightly higher than in the pivotal FUTURE 1 trial (47.4%) (28). Moreover, the European registry study (EuroSpA), which includes over 2,000 patients with PsA treated with secukinumab, reported a remission rate of 39% at 12 months (34), similar to what we observed (46%), and the DAS28-CRP mean change over 12 months was also comparable (−1.1 in both cases). On the contrary, an analysis of the Spanish BIOBADASER registry (two of the 28 participating centers overlap with the present study; *N* = 350) reported a slightly higher 2-year remission rate (77%) but with an almost identical change from baseline (39). Similarly, in a recent real-life study, Molica Colella et al. also observed that, in a population (*N* = 56) that was largely naive to bDMARDs, 68% of patients achieved remission (DAS28-CRP < 2.6) by week 52 of secukinumab treatment (40).

To date, no consensus has been reached on which tool should be used to assess the activity of the disease in patients with PsA or on a clear definition of disease remission (36, 41). DAS28-CRP has often been used as the standard disease activity measure (42), based on the improvements in care and long-term outcomes observed in patients with rheumatoid arthritis (43). However, DAS28-CRP focuses on peripheral arthritis as a domain and includes a limited number of joints. Although it has been shown to correlate with DAPSA (44), most experts agree that the latter is the preferred tool to assess the PsA-disease activity in the clinical setting (41). Despite this, in the present study, DAPSA was recorded only in a reduced number of patients, highlighting the difficulties of applying this index in a busy clinical practice context (45). Nonetheless, in this subgroup of patients, the percentage achieving a DAPSA low disease activity (DAPSA≤14) increased from 27 to 68% after 6 months of treatment with secukinumab, and was maintained up to 24 months (60%), in accordance with the aforementioned DAS28-CRP data. Similarly, in a multicentric study with 608 patients with PsA, Ramonda and colleagues observed a reduction in the mean DAPSA from 25.29 at baseline to 7.69 after 2 years of secukinumab treatment, well within the low disease activity range (46). Adding to our data, Pinto-Tasende et al. showed that after 12 months of secukinumab treatment (*N* = 76), 81.2% of naive and 46.3% of bDMARD-refractory patients achieved very low or low disease activity, according to DAPSA (47).

At baseline, 90% of patients presented active peripheral arthritis. Secukinumab improved peripheral arthritis in a fast and sustained manner as shown by the reduction in the mean TJC and SJC from 6.3 and 3.4 at baseline, respectively, to 3.6 and 1.9 after 24 months of treatment. Our results are in line with the analysis of the Spanish BIOBADASER registry in which the mean baseline TJC and SJC improved from 2.7 and 5.4, respectively, to 0.7 and 1.5 by year 2 of treatment (39). Ramonda and colleagues also demonstrated a clear improvement in TJC and SJC after 6 months of treatment that was maintained for up to 24 months (46). In addition, comparable data were obtained in the observational prospective study AQUILA (*N* = 1,145), which also showed that the efficacy of secukinumab in improving TJC/SJC was similar in males and females (48).

We also analyzed the impact of secukinumab on skin lesions of the disease. Despite not collecting any information regarding the extent of skin involvement, such as surface area or Psoriasis Area and Severity Index (PASI), we observed that after 6 months of treatment 63% of patients with baseline skin involvement had achieved complete skin clearance. Moreover, the percentage of patients with any skin activity by month 24 was greatly reduced. Real-world data on the effectiveness of secukinumab on skin involvement of PsA patients are scarce. Nonetheless, in the AQUILA study 63% of patients achieved a PASI = 0 (skin clearance) at week 52 of treatment (49), similar to the 71% obtained in our study; in both cases it represents an increase of 40% when compared to baseline. Ramonda and colleagues also observed that secukinumab reduced the mean baseline PASI from 4.24 to 0.88 at month 24 (46). In EXCEED, a head-to-head trial in patients with PsA, secukinumab was as efficacious as adalimumab in improving musculoskeletal endpoints, but provided better response on skin endpoints. Thus, the extent of skin involvement might be key when choosing a specific treatment in some patients (19).

In parallel to our data on skin, we observed that the percentage of patients with active nail involvement decreased from baseline to month 24, respectively. Data from a recent interim analysis of the SERENA European registry including 534 patients with PsA treated with secukinumab showed maintenance of a low percentage of patients with active nail involvement up to 2 years under secukinumab treatment (33). Furthermore, our findings reinforce data from the FUTURE 5 clinical trial, which demonstrated an improvement in the modified Nail Psoriasis Severity Index (mNAPSI) of PsA patients with nail involvement up to 2 years of secukinumab treatment (50).

The FUTURE clinical trials showed that secukinumab is effective in treating dactylitis and enthesitis in PsA patients (24, 25, 30, 37, 38). Similarly, we observed a decrease in the percentage of patients with enthesitis from 25% at baseline to 6% at the end of follow-up, showing percentages of complete resolution of enthesitis over 80% from month 6 and thereafter. Dactylitis was active in 20% of patients at baseline and by month 24 that percentage decreased to 4%. As with enthesitis, we observed a 6-month and 24-month complete resolution of dactylitis in about 65 and 80% of patients with baseline dactylitis. These results are similar to recent real-world data from the US-based Corrona registry (*N* = 100) (51) in which more than 60% of patients achieved complete resolution of enthesitis and dactylitis after 6 months of secukinumab treatment. The long-term maintenance of a low percentage of patients with active enthesitis and dactilytis is also supported by the data obtained in the SERENA study (33). In addition, our 2-year data on complete resolution of enthesitis and dactylitis also matches that of the *post hoc* analysis of the FUTURE trials on the efficacy of secukinumab on enthesitis and dactylitis (52, 53).

Psoriatic arthritis can significantly worsen health-related QoL (54). Consequently, pain relief is an essential treatment goal. We observed that secukinumab reduced pain VAS, as well as ptGA and phGA scores. The improvement was detected as early as month 6 and maintained up to month 24 across all three measurements. Similar to our study, Ramonda et al. observed a clear improvement in pain and ptGA after 6 and 24 months (51). In accordance to our data, in the SERENA study was observed a maintenance of pain around 30 mm on a VAS scale up to 2 years of follow-up with 80% of patients achieving a phGA of 0/1 (33). Furthermore, the BIOBADASER registry-based study described that patients with PsA reported a ptGA improvement up to 3 years of secukinumab treatment (39). The above-mentioned AQUILA study also measured the effect of secukinumab on the ptGA and phGA. The observed mean change from baseline to week 52 of treatment was similar to our data, with patients and physicians, respectively, reporting a reduction of about 2.0 and 2.8 points on a 0–10 VAS scale compared to an equivalent 1.4 and 2.2 points in our study (49). Interestingly, in the AQUILA study was also observed a comparable effect on male vs. female patients (55). Of note, in two adicional studies secukinumab was also associated with high levels of patient (56) and physician (57) satisfaction.

Axial PsA has gained increasing attention in the last few years. Despite affecting 25–70% of patients with longstanding PsA, its burden of disease is often underestimated (29), likely because we still lack a clear definition (58). In an effort to do so, ASAS and GRAPPA are leading the AXIS study which aims to develop classification criteria and a unified nomenclature for axial involvement in PsA (59). In our study, about 40% of patients presented active axial disease—as defined by the treating physician—before initiating secukinumab treatment. Unfortunately, our clinical records did not include any measurement for axial disease and therefore were unable to evaluate effectiveness in axial PsA. The last update of the GRAPPA guidelines highlights the need to deeply address this domain as it greatly impacts patients QoL and suggests that IL-17i could be considered for the treatment of axial PsA (60). Specifically, in the MAXIMISE phase IIIb, double-blind, placebo-controlled clinical trial, both the 300 and 150 mg doses of secukinumab significantly improved ASAS20 response vs. placebo at week 12, as well as reduced the total Berlin MRI score for the entire spine and sacroiliac joints (23, 29).

Our findings demonstrated a high probability of secukinumab retention that is consistent with previous reports (34, 39, 40, 46, 49, 61–63). The 12-month persistence rate was very similar to that obtained in EuroSpA study and the BIOBADASER Spanish registry (34, 39). We also observed a high 24-month retention rate of 67%, similar to the retention rate observed by Ramonda et al. (46). By contrast, Alonso et al. observed a lower 24-month retention (43%) which may be related to their population (*N* = 59) being largely refractory to prior biological therapy (61). In our study, lack/loss of efficacy accounted for 80% of all discontinuations while 10% of patients stopped treatment due to adverse events. This is in line with data from BIOBADASER which at its core is a safety registry of bDMARDs. A recent analysis of BIOBADASER showed that the main causes for secukinumab discontinuation were lack of effectiveness (67.9%) followed by adverse events (16.4%) (39).

The generally recommended initial dose of secukinumab is 150 mg. However, patients with concomitant moderate to severe plaque psoriasis or a prior inadequate response to TNFi should initiate treatment with 300 mg (64). Secukinumab dose can be subsequently increased from 150 to 300 mg if disease activity persists, which allows for some dose flexibility. We observed that 33% of naive patients who started with 150 mg required (according to physician criteria) uptitration to 300 mg. Around one third of patients with at least one prior bDMARD failure started with the lower-than-indicated 150 mg secukinumab dose. The reasons behind this choice are unclear and could be varied, but suggest a certain reluctance on the part of some rheumatologists to use full doses from the start. Given that most of these patients (64%) needed an uptitration, this is not an approach to be encouraged. Data from FUTURE 1 and FUTURE 2 clinical trials showed that both secukinumab doses were effective in improving the signs and symptoms of patients with PsA but the 300 mg dose was especially effective in TNFi-experienced patients (24, 25). In both trials, the uptitration to 300 mg improved not only ACR, but also PASI responses as well. In addition, the CHOICE study showed that naive patients with inadequate response to secukinumab 150 mg improved their ACR, enthesitis, dactylitis, and minimal disease activity (MDA) responses after uptitration to secukinumab 300 mg (65). Taken together, clinical trial data and our study suggest that adapting and individualizing the dose of secukinumab might be crucial to achieve the best possible outcome. Finally, 47% of patients initiated secukinumab treatment as monotherapy and 53% in combination with csDMARDs. We observed that during the 2-year follow-up only 9.5% of patients required a modification of monotherapy/combination therapy. Of them, 58.8% switched from receiving secukinumab in combination to monotherapy, while 41.2% experienced the opposite switch (data not shown). Furthermore, the FUTURE 1 and FUTURE 2 trials showed that secukinumab efficacy in joint involvement was similar when used in monotherapy or combined with csDMARDs (27, 28). Altogether, we suspect that the overall impact of concomitant csDMARDs on secukinumab effectiveness and persistence could be mild.

Limitations of this study include the absence of a control group and missing information on several outcomes due to the observational nature of the study. However, most patients had available data for the majority of study parameters. The impact of secukinumab on radiographic progression was not analyzed, despite its importance for patient management. Unfortunately, quantification of radiographic changes is rarely collected in clinical practice. Similarly, we did not collect data about the effectiveness of secukinumab on the axial domain. However, specific measures/outcomes for axial PsA are yet to be defined. We also lack data on the extent of skin and nail involvement. Nonetheless, we were able to measure the percentage of patients achieving complete clearance which is a reasonable treatment goal for both domains. Finally, safety information was limited but among patients that discontinued secukinumab treatment, adverse events only accounted for 10% of them. Individual adverse events were reported through the official channels and were not collected here.

In conclusion, this study expands the knowledge on the use, effectiveness, and persistence of secukinumab in patients with PsA in a real-world setting and complements clinical trial data. We showed that secukinumab is often initiated at a lower dose than recommended and that a significant proportion of patients need an uptritation. Our results demonstrate that secukinumab is able to rapidly improve various domains of PsA thus reducing disease activity. We also report high persistence rates, especially in naive patients. Altogether, our findings support the use of secukinumab as one of the first bDMARDs of choice in regular clinical practice, as recommended in the last update of the GRAPPA international guidelines.

## Data availability statement

The raw data supporting the conclusions of this article will be made available by the authors, without undue reservation.

## Ethics statement

The studies involving humans were approved by the Ethics Committee of the General University Hospital in Elda (Alicante, Spain). The studies were conducted in accordance with the local legislation and institutional requirements. Written informed consent for participation was not required from the participants or the participants’ legal guardians/next of kin in accordance with the national legislation and institutional requirements.

## Author contributions

JA-S: Conceptualization, Formal analysis, Investigation, Writing – original draft, Writing – review & editing, Data curation. VN-M: Writing – review & editing, Data curation, Investigation. CC-F: Writing – review & editing, Data curation, Investigation. IB-T: Writing – review & editing, Data curation, Investigation. MR-V: Writing – review & editing, Data curation, Investigation. MA-Z: Writing – review & editing, Data curation, Investigation. MG-B: Writing – review & editing, Data curation, Investigation. TP-P: Writing – review & editing, Data curation, Investigation. CP-G: Writing – review & editing, Data curation, Investigation. IM: Writing – review & editing, Data curation, Investigation. DB-S: Writing – review & editing, Data curation, Investigation. L-YK: Writing – review & editing, Data curation, Investigation. AC-M: Writing – review & editing, Data curation, Investigation. AM-C: Writing – review & editing, Data curation, Investigation. FN-B: Writing – review & editing, Data curation, Investigation. JS-G: Writing – review & editing, Data curation, Investigation. FS: Conceptualization, Formal analysis, Investigation, Writing – original draft, Writing – review & editing.
