# Supplementary material for: Real-world effectiveness and persistence of secukinumab in the treatment of patients with psoriatic arthritis
Source: Front Med (Lausanne). 2023 Nov 20;10:1294247. doi: 10.3389/fmed.2023.1294247 (PMC10694458; doi:10.3389/fmed.2023.1294247)
Supplement: Supplementary file 1 [file Table_1.DOCX]

Supplementary Material

Real-world effectiveness and persistence of secukinumab in the treatment of patients with psoriatic arthritis

Juan José Alegre-Sancho*, Victoria Núñez Monje, Cristina Campos-Fernández, Isabel Balaguer-Trull, Montserrat Robustillo Villarino, Marta Aguilar-Zamora, Marta Garijo-Bufort, Teresa Pedraz-Penalva, Carolina Peña-González, Isabel de la Morena, Diego Bedoya-Sanchís, Liliya Yankova-Komsalova, Arantxa Conesa-Mateos, Anna Martinez-Cristóbal, Francisco Javier Navarro-Blasco, Jose Miguel Senabre-Gallego, Francisca Sivera

***Correspondence:** Juan José Alegre-Sancho, [alegre_juasan@gva.es](mailto:alegre_juasan@gva.es)

**Supplementary Table 1. Prior treatment**

| **Treatment** |  |
| --- | --- |
| Prior DMARD (yes), n (%) [N] | 166 (93) [178] |
| *Biologic* | 113 (68) [166] |
| Adalimumab | 74 (66) [113] |
| Etanercept | 65 (58) [113] |
| Golimumab | 44 (39) [113] |
| Certolizumab | 21 (19) [113] |
| Infliximab | 20 (18) [113] |
| Ustekinumab | 22 (20) [113] |
| Abatacept | 1 (1) [113] |
| Rituximab | 1 (1) [113] |
| Ixekizumab | 1 (1) [113] |
| Tocilizumab | 0 (0) [113] |
| *Conventional synthetic* | 150 (90) [166] |
| MTX | 138 (92) [150] |
| Leflunomide | 56 (37) [150] |
| Sulfasalazine | 40 (28) [150] |
| Other | 7 (5) [150] |
| *Targeted synthetic* | 10 (6) [166] |
| Apremilast | 8 (80) [10] |
| Tofacitinib | 3 (30) [10] |
| Baricitinib | 0 (0) [10] |

^DMARD: Disease-modifying antirheumatic drug, MTX: methotrexate^
